# Promoting Healthy Lifestyle and Well-Being in Adolescents through Outdoor Physical Activity

**DOI:** 10.3390/ijerph14050533

**Published:** 2017-05-17

**Authors:** Karel Fromel, Michal Kudlacek, Dorota Groffik, Zbynek Svozil, Adam Simunek, Wieslaw Garbaciak

**Affiliations:** 1 Faculty of Physical Culture, Palacký University Olomouc, 771 11 Olomouc, Czech Republic; karel.fromel@upol.cz (K.F.); zbynek.svozil@upol.cz (Z.S.); adam.simunek@upol.cz (A.S.); 2 The Jerzy Kukuczka Academy of Physical Education, 40-065 Katowice, Poland; d.groffik@awf.katowice.pl (D.G.); w.garbaciak@awf.katowice.pl (W.G.)

**Keywords:** sport preferences, health, environment, trends, pedometers

## Abstract

Health-enhancing physical activities (PA) performed outdoors could markedly contribute to the adoption of a healthy lifestyle in adolescence. The differences between PA preferences and actual opportunities for these PA are an issue that has received frequent attention. To date, the extent to which these differences are reflected in adolescents meeting PA recommendations and their well-being has not been explored. In total, 10,086 respondents took part in an on-line research project regarding PA preferences. Of them, 2446 also completed the International Physical Activity Questionnaire (Long Form) and the World Health Organization (WHO) W-5 questionnaire to assess well-being. Finally, 1278 of these respondents were involved in objective PA monitoring using pedometers. The study aimed to explore the prevalence and trends regarding outdoor PA. Moreover, we assessed whether the agreement between preferred PA and PA actually undertaken was associated with higher odds for meeting PA recommendations and achieving a higher level of well-being. Of a selection of outdoor activities, Czech and Polish boys preferred cycling, swimming, and downhill skiing, while girls preferred swimming activities, skating, and cycling. The agreement between preferred and PA actually undertaken was associated with higher odds for meeting the weekly PA recommendations and higher levels of well-being both in boys and girls. Evaluation of outdoor PA preferences and taking these preferred activities into account when forming conditions for them was important in the efficient promotion of the physical and mental health of adolescents.

## 1. Introduction

The idea of a ‘return to nature’, has been cyclically recurring in various schools of philosophy throughout recorded history, for example the Ancient Greek cynicism defined by Antisthenes [1], Rousseaus’ approach to education in the age of enlightenment [2], or Husserl’s phenomenological interpretation of nature in science [3]. The philosophical trend of a return to nature has a significant ecological, demographic, health-related, and economic impact. Active and health-enhancing leisure time by outdoor physical activities (PA) can significantly increase the benefits of PA and facilitate adoption of a healthy lifestyle in children and adolescents, and can also be combined with the school environment. Different forms of natural environment (parks, meadows, woods) are associated with a feeling of happiness [4], a decrease in mental stress [5,6], and can significantly affect emotional well-being [7]. Repeated exercises in the natural environment promote well-being more than exercise in the built environment, regardless of whether they are performed indoors or outdoors [7,8]. Access to the natural environment for PA should, therefore, be protected and supported to enhance the mental health of a population [9,10].

The ability to perform PA in the natural environment (nature awareness, green thinking) could be one of the key factors that could reduce the negative impact of decreasing levels of PA and increasing levels of sedentary behavior [11,12,13,14]. However, there could be eventual negative effects of large scale organized PA in nature on the environment [15,16], and these negative effects need to be eliminated to the maximum possible extent. Indeed, we need to exploit the positive effects of outdoor PA to change the lifestyle of adolescents, because experiences gained in nature are much more effective in promoting a healthy lifestyle than presenting facts regarding negative health trends.

PA researchers still tend to focus more on volume, intensity, duration, and frequency of PA than on the type of PA. Stenley et al. [17] analyzed the most common types of activities during lunch breaks and after-school time among Australian pupils. They found that 57% of the most frequent activities during the after-school period were sedentary, while only 43% involved moderate-to-vigorous PA.

However, much less attention is devoted to the evaluation of PA preferences in the context of environmental settings. It appears that environment has and will continue to have an increasing influence on the promotion of healthy lifestyles across all population groups, including children and adolescents [5,18]. Research into the environment of physical activity devotes most attention to the built environment [19,20], parks and urban green spaces [21,22,23], the urban environment [24], and the less typical natural environment outside urban agglomerations [25,26].

Investigation of differences between PA preferences and actual opportunities for doing these preferred activities therefore becomes important. Diagnostics of the PA preference structure should be an undoubtedly important component to improve the current situation regarding prevalence of PA and health lifestyle [27,28,29]. Identification of the preferred activities among children and adolescents is important especially because various activities can enhance different cognitive, social and motor skills and moreover because these gender-related issues change with age [30]. Thus far, there has been little research on the extent to which these differences are reflected in adolescents meeting PA recommendations and their well-being, which both constitute substantial attributes of their physical and mental health. 

The prevalence of physical inactivity is the essential problem in the Czech Republic, as well as in the majority of the countries in the world [31]. That is why we put emphasis on adolescents’ sport preferences in outdoor settings research in the context with opportunities for their meeting.

This study aimed to explore the prevalence and trends regarding outdoor PA. Moreover, we assessed whether the agreement between preferred and PA actually undertaken was associated with higher odds for meeting PA recommendations and showing higher levels of well-being. 

## 2. Methods

### 2.1. Participants and Settings

**Figure 1:**
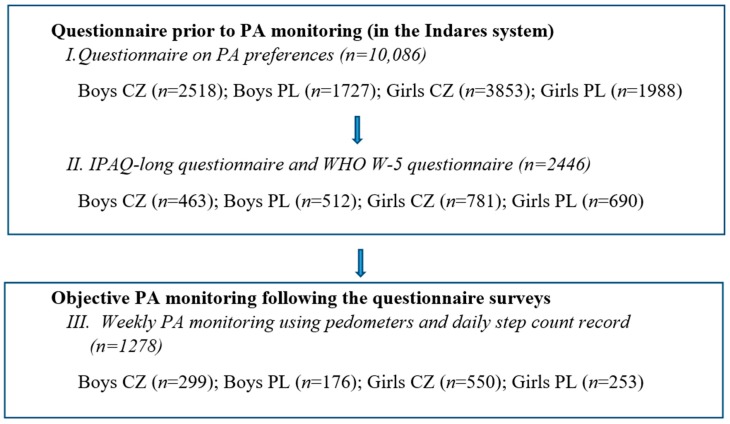
Research design in Czech (CZ) and Polish (PL) boys and girls. PA: physical activity; Indares: Internet program; IPAQ: International Physical Activity Questionnaire: WHO: World Health Organization.

In total, 10,086 adolescents (boys *n* = 4245, age 16.72 ± 1.44, weight 69.07 ± 11.68, height 177.92 ± 7.95, body mass index (BMI) 21.75 ± 2.99; girls *n* = 5841, age 16.87 ± 1.43, weight 58.22 ± 8.83, height 167.09 ± 6.31, BMI 20.83 ± 2.74) took part in the online research on PA preferences between 2009 and 2016. The research was conducted at 3 levels differing in their scale according to the consent given by school authorities. On average, 93% of addressed students agreed to participate in the research. The participants from 248 schools (157 Czech Republic, 91 Polish) were registered in the International Database for Research and Educational Support (Indares) (www.indares.com) and completed the questionnaire on PA preferences. The next research tier was conducted in 115 schools (55 Czech Republic, 60 Polish) and 2446 participants also completed the International Physical Activity Questionnaire (long form) (IPAQ-LF) and the World Health Organization (WHO) W-5 to assess their well-being. In the final tier, 1278 participants from 51 schools (32 Czech Republic and 19 Polish) took part in a weekly PA monitoring study using pedometers (Figure 1). The ethics approval was obtained from the Institutional Research Ethic Committee of Palacký University (ID: no. 24/2012) and voluntary informed consent was provided from all participants.

### 2.2. Measurements

#### 2.2.1. Physical Activity Preferences

PA preferences were assessed using the questionnaire on PA preferences, which is a standardized questionnaire [32,33]. The types of PA preferences were divided into 8 categories: individual PA, team PA, fitness-related PA, water-based PA, outdoor PA, martial arts, rhythm and dance PA, and overall PA. The categories of outdoor PA preferences were as follows:

1. Board sports (skateboard, surfing, kiting)
2. Skating (in-line, roller skates)
3. Cycling
4. Golf
5. Horse riding
6. Rope activities
7. Aviation, gliding, hang-gliding
8. Climbing (rock climbing, wall climbing)
9. Boat activities (rafting, kayaking, canoeing, yachting)
10. Cross-country skiing
11. Downhill skiing, skialpinism
12. Motor sport, skiering, water motor sport
13. Orienteering (radio, skiing)
14. Parachuting (paragliding, skydiving, airboarding)
15. Hiking, snowshoeing, tramping
16. Swimming, water activities
17. Snowboarding

The participants selected the top 5 preferred PA from the list (or mentioned a PA of a similar type). The selected PA then received points for their ranking. The unselected PA were given points based on the average of the other rankings, which was 11.5 points in this category. The ranking of preferred outdoor PA was determined by the sum of points for ranking and subsequently the average number of points given for ranking. The questionnaire also contained items focusing on participation in organized PA in the course of the year, investigating the number of hours per week spent in the given PA, and the most frequently performed PA in the summer and winter seasons. According to the preferred and actually performed PA we determined the key independent variable; that is, the agreement between the preferred PA and the PA actually undertaken that was listed in the top 5 preferred activities by a respective respondent. Trends in preferred outdoor PA were investigated in 2-year intervals between 2009 and 2016.

#### 2.2.2. Subjective Estimate of Weekly PA

To assess weekly PA before starting to monitor PA by pedometers, we used the online Czech and Polish versions of the IPAQ-LF in the Indares system [34]. Both the versions were subject to the translation procedure according to the European Organisation for Research and Treatment of Cancer (EORTC) Quality of Life Group [35]. The IPAQ-LF questionnaire covers various types of PA (job/school-related PA; transportation PA; housework, house maintenance, and caring for family PA; recreation, sport, and leisure-time PA), various intensities of PA (vigorous, moderate, and walking), and time spent sitting. We wanted to avoid overestimation of the time spent in PA and underestimation of time spent being sedentary, and thus, maintain the objective composition of PA. To this end we proceeded as follows: (a) In contrast to the IPAQ-LF questionnaire handbook, we multiplied metabolic equivalent (MET)-minutes of vigorous PA by 6 and not by 8 as recommended; (b) The estimated minutes of weekly PA in particular types of PA were transformed to average daily minutes of PA; (c) The maximum daily sum of PA and transportation PA was set at 600 min. The maximum amount of MET-minutes per week was set at 20,000 MET-minutes. Because they failed to meet the given criteria, 264 participants were excluded from the analyses [36].

As a recommendation of weekly PA, we used the suggested minimum amounts of PA, amended in line with Healthy People 2010 [37], Healthy People 2020 [38], 2008 Physical Activity Guidelines for Americans [39] and European Union (EU) Physical Activity Guidelines [40]. The minimum values were used because meeting the PA recommendation according to the IPAQ-LF was based only on a single PA of a given kind. The minimum recommendation for vigorous physical activity (VPA) was to achieve at least 3 or more days per week for 20 or more minutes. For general PA, regardless of intensity, the minimum was at least 5 or more days per week for 60 min, and the simultaneous meeting of both the PA recommendations (5 × 60 min of PA + 3 × 20 min of VPA).

#### 2.2.3. Objective PA Monitoring Using Pedometers

The Yamax Digiwalker SW-700 (Yamax Corporation, Tokyo, Japan) pedometer was used for objective monitoring of weekly PA. This type of pedometer is suitable for the assessment of habitual PA expressed as step count [41]. The extreme values of pedometer-derived data were adjusted in line with Tudor-Locke [42]. Data for any single day indicating less than 1000 steps were removed and values greater than 30,000 steps on any single day were truncated to 30,000 steps. The participants had to have a valid record from at least 3 school days and 1 weekend day. The data for any missing data were replaced by the very next school or weekend day. Overall, 151 participants were excluded and 116 values of daily step count were inserted into the dataset. The PA recommendation of 11,000 steps/day was based on the recommended dimensions of daily step count in line with Tudor-Locke et al. [43], but it was the same for boys and girls. To reduce reactivity of PA monitoring, the participants wore the pedometers immediately after the initial training session [44]. The actual weekly monitoring was launched on the following day and lasted for a week. The participants wore the pedometers from the morning (after personal hygiene) for the entire day (except for swimming or bathing) until going to bed (evening hygiene).

#### 2.2.4. Self-Reported Well-Being

To assess the well-being index, we used the W-5 Czech and Poland questionnaire (https://www.psykiatri-regionh.dk/who-5/Pages/default.aspx). WHO-Five Well-Being Index is a valid screening instrument for children and adolescents in pediatric care [45,46].

#### 2.2.5. Healthy Lifestyle

Concurrently achieving weekly PA on at least 5 or more days per week for 60 min and at the same time vigorous PA on 3 or more days per week for 20 or more minutes (5 × 60 min PA + 3 × 20 min VPA) and exceeding the cut-off point for well-being (≥13 points) represented a narrowly defined indicator of a healthy lifestyle.

#### 2.2.6. Data Analysis

We used the descriptive statistics, crossing tables, Kruskal-Wallis test—nonparametric alternative to one-way analysis of variance, logistic regression and η^2^ and w effect size coefficients [47,48]. The essential matching between preferred PA and PA actually undertaken was done via IPAQ-LF (PA actually undertaken) with consequential analysis of the results acquired from the questionnaire on PA preferences. The data was analyzed using the Statistica version 13 (StatSoft, Prague, Czech Republic) and SPSS version 22 (IBM, Chicago, IL, USA) programs. 

## 3. Results

### 3.1. Ranking of Preferred Outdoor PA

Czech and Polish boys preferred cycling, swimming, and downhill skiing, while girls preferred swimming activities, skating, and cycling (Table 1). The biggest differences between Czech and Polish boys, as well as between girls, were found in preferences for rope activities. Czech and Polish girls preferred horse riding, while boys preferred motor sports.

### 3.2. Trend in Preferred Outdoor PA

**Figure 2:**
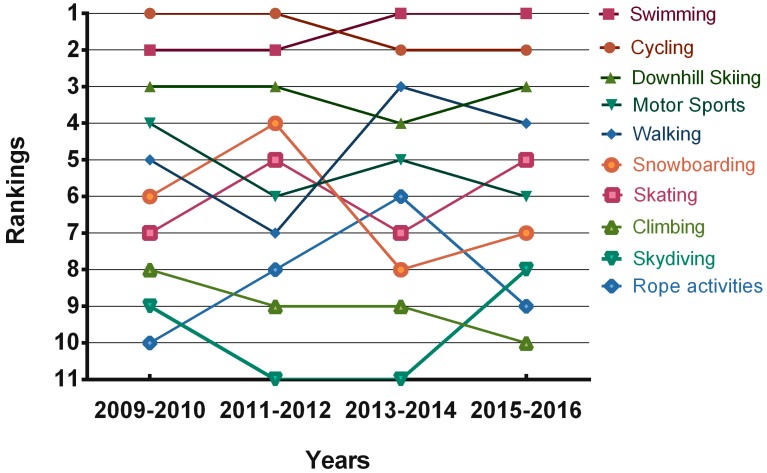
Outdoor PA preferences in boys between 2009 and 2016.

**Figure 3:**
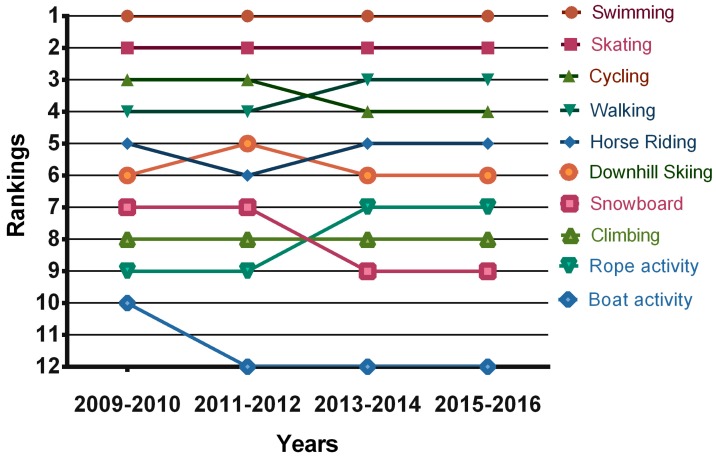
Outdoor PA preferences in girls between 2009 and 2016.

The multi-year analysis of outdoor PA showed stability regarding the preference of cycling, swimming activities, and downhill skiing among boys (Figure 2) and swimming activities, skating, and cycling among girls (Figure 3). From the long-term perspective, a greater stability is apparent in outdoor PA preferences in Czech and Polish girls than in boys from both the countries.

The most preferred outdoor activities (those ranked first) in boys were cycling (160 times), swimming (120 times), and downhill skiing (101 times). In girls, skating (270 times), swimming (207 times), and horse riding (171 times) ranked highest.

### 3.3. Achievement of PA Recommendations and Higher Levels of Well-Being

**Figure 4:**
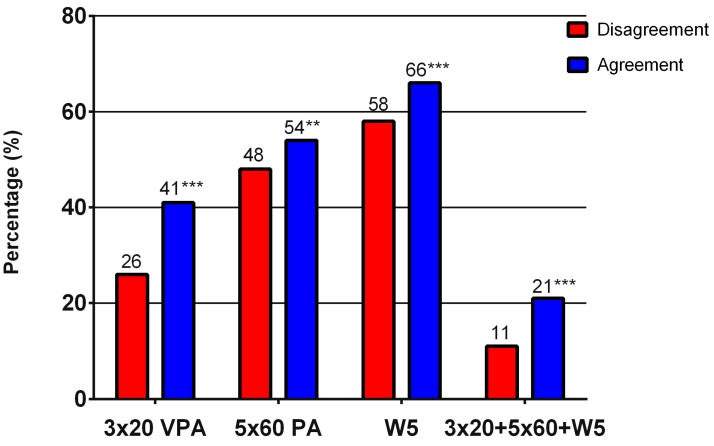
Meeting recommendations for physical activity and reporting well-being in boys and girls, stratified by agreement in preferred and actually performed PA (indicated as Agreement). ** statistical significance of *p* < 0.01; *** statistical significance of *p* < 0.001; VPA: vigorous physical activity; W5: WHO-Five Well-Being Index.

Of adolescents who preferred the same PA as the one they actually took part in, a significantly larger number of individuals met the minimum recommendation for vigorous PA (Pearson’s chi-square, χ^2^ = 52.49, *p* ˂ 0.001; Cohen’s effect size index w = 0.146*) and the minimum recommendation for overall PA (χ^2^ = 9.08. *p* = 0.003; w = 0.061); they reported higher levels of well-being (χ^2^ = 17.51, *p* ˂ 0.001; w = 0.085); and met the PA recommendation while reporting higher well-being simultaneously (χ^2^ = 33.53, *p* ˂ 0.001; w = 0.117*), than those who did not engage in their preferred activities (Figure 4).

**Figure 5:**
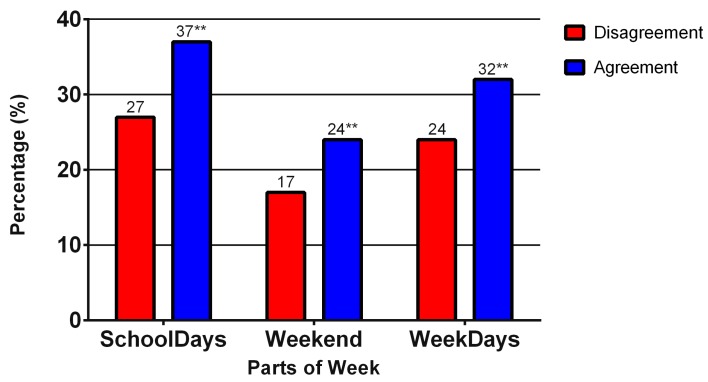
Meeting recommendations for daily step count (11,000 steps/day), stratified by agreement between preferred and actually performed PA and higher well-being of participants (indicated as Agreement). ** statistical significance of *p* < 0.01; *** statistical significance of *p* < 0.001.

Similarly, adolescents who show the agreement between preferred and actually performed PA and higher well-being met the daily step count recommendation (11,000 steps/day) on school days (χ^2^ = 10.26, *p* = 0.001; w = 0.090), weekend days (χ^2^ = 7.84, *p* = 0.005; w = 0.085), and throughout the week (χ^2^ = 7.65, *p* = 0.006; w = 0.077) significantly more often than those who lacked this agreement (Figure 5). However, low values of the w effect size coefficient indicated a low practical significance of the differences observed.

Significant differences in achieving the PA recommendations according to the IPAQ-LF questionnaire and simultaneously higher levels of well-being were observed regarding the following independent variables: sex (boys 21.3% vs. girls 12.6%), countries (Polish 19.6% vs. Czech Republic 13.9%) and size of city (23.1% of adolescents from large cities vs. 11.3% of those from rural areas) (Table 2). We observed the difference by BMI (30.4% of adolescents with normal weight vs. 21.4% of those overweight/obese) regarding the daily step count recommendation and reporting higher levels of well-being.

Other observed variables with the potential to influence the level of PA were ownership of a dog (by family) and type of living (house, flat). Ownership of a dog was observed in the research of adolescents’ walking, which found that dog walkers had 7–9% more min/day of moderate to vigorous physical activity (MVPA; intensity of recommended PA by national and international public health authorities) than ‘non-dog walkers’ [49]. Similarly, Sirard et al. [50] confirms that dog ownership was associated with more PA among adolescents. Type of living is considered a significant attribute of socio-economic status in the Czech Republic [51], which is why we included this variable among observed variables.

Agreement between preferred and PA actually undertaken increased the odds for meeting PA recommendations and reporting higher levels of well-being (Table 3). The odds were not reduced by adjusting model 2 for gender and other variables, and model 3 for gender, size of city, and other variables. 

In addition, an agreement between preferred and PA actually performed confirmed an increase of odds for meeting the daily step count recommendations and reporting higher well-being (Table 4). The odds were not reduced by adjusting model 2 for BMI and other variables, and model 3 for BMI, countries, and other variables.

## 4. Discussion

The key finding of the present study is that adolescents showing agreement between preferred PA and activities actually performed met the PA recommendation more often than adolescents who did not show this agreement. Other findings regarding such associations have not been published yet. There is no evidence describing and testing the conformity of PA preferences and PA actually performed with its consequences on well-being, and on health generally.

Analysis of preferred outdoor physical activities clearly displays the dominating role of swimming and water-based activities and cycling concerning both gender and trend. We found no significant differences between Czech and Polish adolescents in PA preferences, which is in line with the findings of Křen [32] and Neuls [52]. Brown et al. [53] reached similar conclusions in their research of activity preferences in school-aged children in rural and urban locations. There were no significant differences in the participation patterns of boys and girls. The biggest differences in outdoor PA preferences between girls and boys involved in our study were observed in skating (girls preferred this activity more often) and downhill skiing (boys preferred this activity more often).

On the other hand, Lee [54] showed significant differences in preferences by gender; boys preferred moderate-to-vigorous PA in the form of team sports such as football, basketball, and soccer, while girls preferred moderate individual PA such as dancing, cycling, skating, and walking. Both studies were conducted in different cultural settings, which could be the explanation for this variability within results. These results could also serve as an indicator of diminishing gender specifics within central European countries, particularly within the Czech Republic and Poland. Work by Martinez-López et al. [55] also highlighted the importance of acknowledging gender issues with regard to the associations between well-being and PA. They state that low levels of weekly PA are associated with an increased risk of pain and a decrease in teenagers' well-being, and this occurs to a different extent for boys and girls.

The importance of gender differences exploration is significant also from various educational systems all around the world. Adolescents’ preparation and motivation for leisure-time PA is significantly different at schools with co-educative or differentiate physical education (a major part of the countries in middle and east Europe).

It is reasonable to take educational, socioeconomic, demographic, ethic, and other characteristics into account when analyzing gender differences in PA preferences. In this context, Eime et al. [56] pinpointed the importance of organized PA in their Conceptual Model of Health through Sport. This model was conceived at 2 levels addressing various aspects of physical activities: (1) individual/team nature of activities, (2) organized/unorganized activities. We agree with the design of the model or, more precisely, the necessity to consider these characteristics. However, because of the complex design of our study, we did not address these aspects. 

We focused on outdoor physical activities intentionally, because of the positive aspects of the natural environment. Physical activities in the natural environment influence both the level of fitness in a population, particularly mental health and general life satisfaction [57,58]. Compared with physical activities undertaken in other environments, outdoor physical activities contribute significantly to higher well-being [8,21]. The benefits of outdoor physical activities for the enhancement of mental and social health were also supported by work by Thompson Coon et al. [8]. Unfortunately, they did not focus on the associations with physical health and well-being. This is the first study examining the associations of objectively measured PA in relation to preferred activities and well-being.

Objective PA monitoring by pedometers was included as a more objective indicator of PA level to complement the subjective estimate of weekly PA using the IPAQ-LF. The natural environment was shown as a stimulating factor for meeting the daily step count recommendation (11,000 steps/day) [43]. The participants showing the agreement between preferred and PA actually performed have higher odds of reaching 11,000 steps/day. The estimate of PA by monitoring the daily step count is suitable for the natural environment due to its ultimate nature and principles. This emphasizes the explorative dimension, together with another fundamental stimulus typical for the natural environment—getting from one place to another [7]. The beneficial effect of the natural environment on PA level, and in turn, on health has also been described by Coombes [59].

The research on PA in the natural environment by using pedometers also has barriers and methodological issues, including gender issues. A study by Vasickova [60] showed that girls respond to pedometer wearing more positively than boys. This can significantly contribute to the equalization of opportunities for leisure-time PA in research, but also in PA beyond the research surveys. This work was confirmed by Ho [61], which illustrates the need to take gender issues into account when interpreting the results of outdoor PA monitoring.

We believe that differences in the level of PA between girls and boys, as well as the consequences of an insufficient amount of PA, are determined by the actual type of PA; that is, differences between preferred and performed PA. As research on preferences regarding PA still represents a certain research gap, we recommend that this topic is studied systematically in future research.

The most important practical implications for real-life settings is respecting gender differences and variability in PA preferences, as well as exploring and identifying the trends of preferred and actually realized physical activities. This knowledge has potential to create conditions for adequate PA, school programs (educational, physical activity), community programs for the improvement of people’s lifestyle or programming activities within various sports organizations. The same attention should be given to the usage of facts about status and trends in preferred PA, actually realized PA and organized PA (structured PA) in children and youth, which will enable us to create effective and functional health, school, environmental and communal policies. 

### Strengths and Limitations

The main strength of the present study is the assessment of associations of agreement between preferred and PA actually performed and achieving PA recommendations and higher levels of well-being in adolescents. The time-series data in outdoor PA preferences in the Central European environment are also important.

A limitation of this study was its cross-sectional design, which makes the association analysis more difficult and hinders the generalizability of the results. Moreover, we did not evaluate the particular type of the natural environment and its influence on well-being and rates of meeting PA recommendations.

## 5. Conclusions

Evaluating outdoor PA preferences and taking the preferred activities into account when forming opportunities for PA are efficient ways to promote the physical and mental health of adolescents and a healthy and physically active lifestyle. From the global perspective, it is important to monitor prevalence and trends in preferred types of outdoor PA with those actually performed.

## Figures and Tables

**Table 1:** Preferences for outdoor physical activity (PA) in boys and girls from the Czech Republic and Poland.

Physical Activities	Boys CZ (*n* = 2518)	Ranking M	Boys PL (*n* = 1727)	Ranking M	Girls CZ (*n* = 3853)	Ranking M	Girls PL (*n* = 1988)	Ranking M
Cycling	1	7.14	1	6.97	3	7.65	3	6.92
Swimming, water attractions	2	7.32	2	7.31	1	5.92	1	5.95
Downhill skiing	3	8.02	3	8.45	4	8.14	7	9.23
Skating	4	8.37	7	9.22	2	6.12	2	6.55
Snowboarding	5	8.61	9	9.37	7	8.53	9	9.75
Hiking	6	8.79	4	8.51	5	8.29	4	7.14
Motor sport etc.	7	8.83	5	8.67	16	10.52	11	10.17
Climbing	8	9.21	8	9.29	8	9.15	8	9.33
Parachuting etc.	9	9.35	10	9.76	10	9.92	12	10.3
Golf	10	9.36	14	10.03	14	10.31	15	10.56
Rope activities	11	9.4	6	9.08	9	9.59	5	8.79
Board sports	12	9.59	15	10.17	12	10.19	13	10.48
Aviation	13	9.62	12	10.02	13	10.26	14	10.55
Cross-country skiing	14	9.89	16	10.29	15	10.36	16	10.66
Boat activities	15	9.94	11	9.99	11	10	10	10.13
Horse riding	16	10.23	13	10.02	6	8.43	6	8.93

M: mean; CZ: Czech Republic; PL: Poland.

**Table 2:** Descriptive characteristics (*n* = 1278).

Variables	Total *n* (%)	3 × 20 Min VPA, 5 × 60 Min PA Recommendation and Higher Well-Being			Steps at Recommended Level of 11,000 Steps/Day and Higher Well-Being		
		Yes *n* (%)	No *n* (%)	*p* Value	Yes *n* (%)	No *n* (%)	*p* Value
***Gender***							
Boys	475 (37.2)	101 (21.3)	374 (78.7)	<0.001 ***	115 (24.2)	360 (75.8)	0.171
Girls	803 (63.8)	101 (12.6)	702 (87.4)		168 (20.9)	635 (79.1)	
***Age (years)***							
15	339 (26.5)	62 (18.3)	277 (81.7)	0.323	101 (29.8)	238 (70.2)	0.653
16	496 (38.8)	72 (14.5)	424 (85.5)		151 (30.4)	345 (69.6)	
17	443 (34.7)	68 (15.3)	375 (84.7)		123 (27.8)	320 (72.2)	
***BMI***							
<25.0	1124 (87.9)	179 (15.9)	945 (84.1)	0.752	342 (30.4)	782 (69.6)	0.021 *
≥25.0	154 (12.1)	23 (14.9)	131 (85.1)		33 (21.4)	121 (78.6)	
***Countries***							
Czech Republic	849 (66.4)	118 (13.9)	731 (86.1)	0.009 **	263 (31.0)	586 (69.0)	0.071
Poland	429 (33.6)	84 (19.6)	345 (80.4)		112 (26.1)	317 (73.9)	
***City (inhabitants)***							
<1000	337 (26.4)	38 (11.3)	299 (88.7)	0.001 **	94 (27.9)	243 (72.1)	0.658
1000–29,999	443 (34.7)	62 (14.0)	381 (86.0)		137 (30.9)	306 (69.1)	
30,000–100,000	290 (22.7)	54 (18.6)	236 (81.4)		88 (30.3)	202 (69.7)	
˃100,000	208 (16.3)	48 (23.1)	160 (76.9)		56 (26.9)	152 (73.1)	
***Home***							
Flat	524 (41.0)	86 (16.4)	438 (83.6)	0.620	152 (29.0)	372 (71.0)	0.826
House	754 (59.0)	116 (15.4)	638 (84.6)		223 (29.6)	531 (70.4)	
***Ownership of a dog***							
No	641 (50.2)	96 (15.0)	545 (85.0)	0.415	184 (28.7)	457 (71.3)	0.616
Yes	637 (49.8)	106 (16.6)	531 (83.4)		191 (30.0)	446 (70.0)	
***Ownership of a car (by family)***							
No	439 (34.3)	73 (16.6)	366 (83.4)	0.560	123 (28.0)	316 (72.0)	0.452
Yes	839 (65.7)	129 (15.4)	710 (84.6)		252 (30.0)	587 (70.0)	

* statistical significance of *p* < 0.05; ** statistical significance of *p* < 0.01; *** statistical significance of *p* < 0.001; VPA: vigorous physical activity; BMI: body mass index.

**Table 3:** Odds ratios for meeting the 3 × 20 min VPA, 5 × 60 min PA recommendation and higher well-being by agreement between preferred and actually performed physical activities.

Variables	Model 1		Model 2		Model 3	
	OR (95% CI)	*p*	OR (95% CI)	*p*	OR (95% CI)	*p*
***Agreement between preferred and actually performed PA***						
**No (ref.)**						
**Yes**	2.593 (1.714–3.924)	<0.001	2.536 (1.672–3.847)	<0.001	2.519 (1.65–3.834)	<0.001
***Gender***						
Girls (ref.)						
Boys			1.877 (1.380–2.553)	<0.001	1.855 (1.357–2.537)	<0.001
***BMI***						
≥25.0 (ref.)						
<25.0			0.896 (0.553–1.453)	0.657	0.894 (0.547–1.460)	0.655
***Age (years)***						
15 (ref.)						
16			0.762 (0.522–1.112)	0.159	0.973 (0.636–1.487)	0.898
17			0.841 (0.572–1.235)	0.376	0.736 (0.493–1.099)	0.134
***Countries***						
Poland (ref.)						
Czech Republic					0.787 (0.538–1.151)	0.217
***City (inhabitants)***						
<1000 (ref.)						
1000–29,999					1.414 (0.886–2.255)	0.146
30,000–100,000					1.983 (1.184–3.322)	0.009
>100,000					2.421 (1.415–4.144)	0.001
***Home***						
House (ref.)						
Flat					0.846 (0.590–1.212)	0.362
***Ownership of a dog***						
No (ref.)						
Yes					0.760 (0.548–1.054)	0.100
***Ownership of a car***						
Yes (ref.)						
No					0.960 (0.687–1.342)	0.813

*OR*: Odds ratio; *CI*: Confidence interval; *p* = statistical significance; Model 1: agreement between preferred and actually performed physical activities; Model 2: Adjusted for gender, BMI and age; Model 3: Adjusted further for countries, city, house, ownership of dog and ownership of car; ref.: reference category.

**Table 4:** Odds ratios for meeting the 11,000 steps/day recommendation and higher well-being by agreement between preferred and actually performed physical activities.

Variables	Model 1		Model 2		Model 3	
	OR (95% CI)	*p*	OR (95% CI)	*p*	OR (95% CI)	*p*
***Agreement between preferred and actually performed PA***						
No (ref.)						
Yes	1.485 (1.121–1.968)	0.006	1.463 (1.103–1.940)	0.008	1.474 (1.109–1.958)	0.007
***Gender***						
Girls (ref.)						
Boys			0.975 (0.758–1.254)	0.845	0.953 (0.738–1.229)	0.709
***BMI***						
≥25.0 (ref.)						
<25.0			1.565 (1.040–2.356)	0.032	1.617 (1.071–2.442)	0.022
***Age (years)***						
15 (ref.)						
16			1.053 (0.777–1.425)	0.740	1.040 (0.765–1.487)	0.802
17			0.939 (0.686–1.286)	0.697	0.820 (0.585–1.150	0.134
***Countries***						
Poland (ref.)						
Czech Republic					1.424 (1.045–1.941)	0.025
***City (inhabitants)***						
<1000 (ref.)						
1000–29,999					0.975 (0.627–1.518)	0.912
30,000–100,000					1.131 (0.759–1.685)	0.545
>100,000					1.211 (0.811–1.808)	0.349
***Home***						
House (ref.)						
Flat					0.984 (0.737–1.314)	0.362
***Ownership of a dog***						
No (ref.)						
Yes					0.877 (0.677–1.137)	0.321
***Ownership of a car***						
Yes (ref.)						
No					0.985 (0.754–1.287)	0.913
